# ROS-DRP1-mediated excessive mitochondrial fission and autophagic flux inhibition contribute to heat stress-induced apoptosis in goat Sertoli cells

**DOI:** 10.1186/s40104-025-01180-2

**Published:** 2025-04-16

**Authors:** Fei Wen, Jiajing Gao, Guoyu Zhang, Songmao Guo, Xing Zhang, Shuaiqi Han, Xianzou Feng, Xiaoxu Chen, Jianhong Hu

**Affiliations:** College of Animal Science and Technology, Northwest Agriculture and Forestry University, No. 22 Xinong Road, Yangling, Shaanxi 712100 People’s Republic of China

**Keywords:** Apoptosis, Goat Sertoli cells, Heat stress, Melatonin, Mitochondria, Mitophagy

## Abstract

**Background:**

Heat stress (HS) poses a significant threat to male goat reproduction. Sertoli cells (SCs) provide both structural and nutritional support necessary for germ cells. HS induces physiological and biochemical changes in SCs. Nevertheless, the molecular mechanisms involved are still not fully understood. Melatonin is a classic antioxidant that can alleviate HS-induced male reproductive damage. However, the underlying molecular mechanisms by which melatonin mitigates damage to goat testicular SCs remain unclear and require further investigation.

**Results:**

In this study, an in vivo heat stress model was established in goats. The results showed that HS exposure led to testicular injury, abnormal spermatogenesis and apoptosis of SCs. To elucidate the mechanism of HS-induced SC apoptosis, primary SCs were isolated and cultured from goat testes, then exposed to HS. HS exposure increased the production of reactive oxygen species (ROS), decreased adenosine triphosphate (ATP) synthesis, and reduced mitochondrial membrane potential in SCs. Additionally, HS increased the expression of mitochondrial fission proteins 1 (FIS1) and dynamin-related protein 1 (DRP1) while decreasing the expression of mitochondrial fusion proteins Mitofusin 1 (MFN1), Mitofusin 2 (MFN2), and optic atrophy 1 (OPA1). This resulted in excessive mitochondrial fission and mitochondria-dependent apoptosis. Mdivi-1 (DRP1 inhibitor) reduces mitochondria-dependent apoptosis by inhibiting excessive mitochondrial fission. Mitochondrial fission is closely related to mitophagy. HS activated upstream mitophagy but inhibited autophagic flux, disrupting mitophagy and exacerbating mitochondria-dependent apoptosis. Finally, the classical antioxidant melatonin was shown to reduce mitochondria-dependent apoptosis in SCs exposed to HS by decreasing ROS levels, restoring mitochondrial homeostasis, and normalizing mitophagy.

**Conclusions:**

In summary, these findings indicated that the mechanism of HS-induced mitochondria-dependent apoptosis in SCs is mediated by hyperactivation of the ROS-DRP1-mitochondrial fission axis and inhibition of mitochondrial autophagy. Melatonin inhibited HS-induced mitochondria-dependent apoptosis in SCs by restoring mitochondrial homeostasis. This study enhances the understanding of the mechanisms through which heat stress triggers apoptosis and provides a vision for the development of drugs against HS by targeting mitochondria in goats.

**Graphical Abstract:**

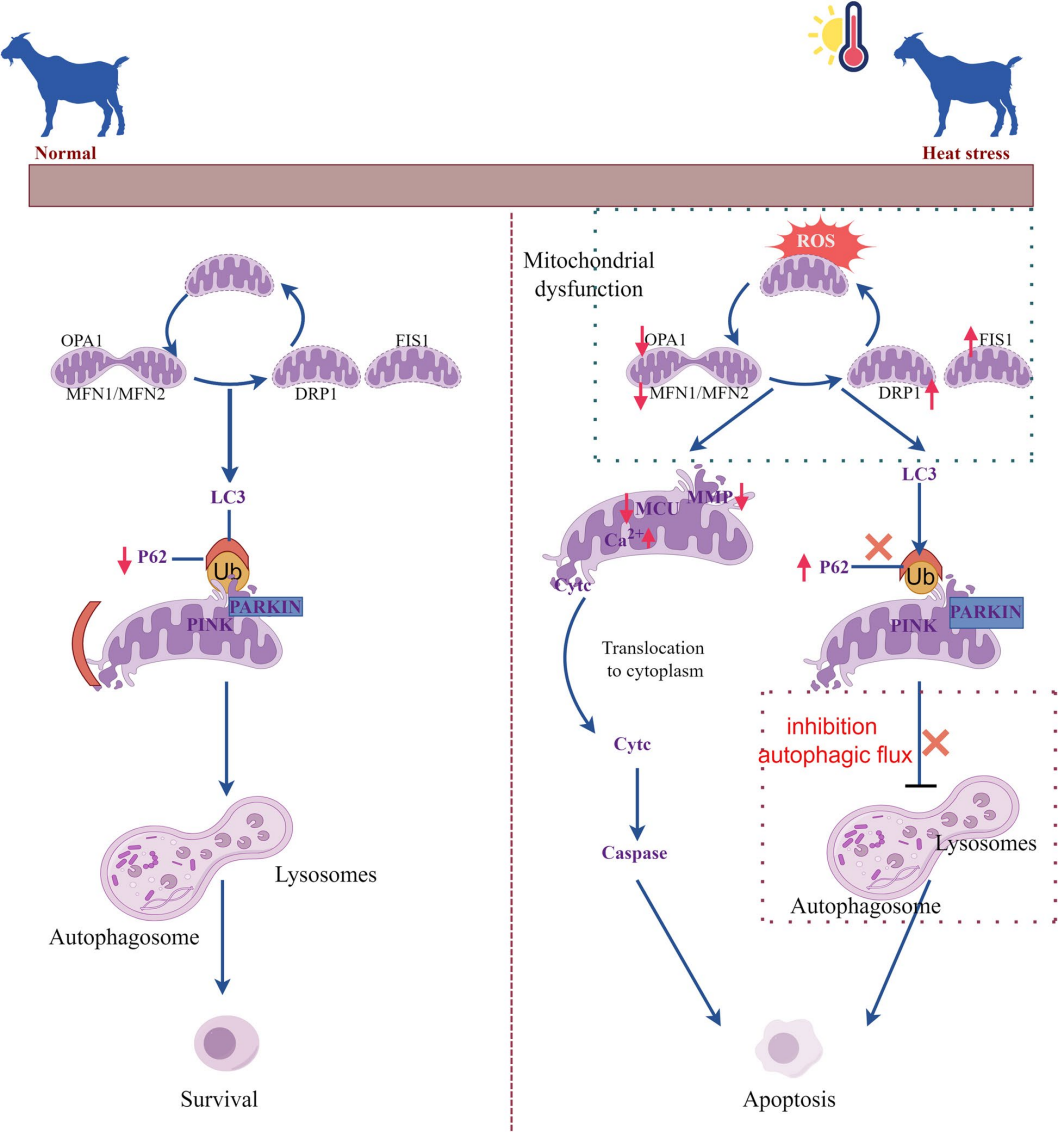

**Supplementary Information:**

The online version contains supplementary material available at 10.1186/s40104-025-01180-2.

## Background

Global surface temperatures have been rising steadily since the latter half of the twentieth century [1]. Livestock have a limited ability to adapt to temperature changes. Heat stress (HS) caused by high temperatures can reduce growth and lower reproduction rates, thereby compromising dairy production and quality. This, in turn, directly impacts the economics and sustainability of livestock farming [2, 3]. In male animals, HS can induce oxidative stress and inflammatory responses in the body, thereby impairing the testicular cell function and spermatogenesis [4, 5]. Numerous studies have shown that HS results in reduced numbers of germ cells, leading to impaired spermatogenesis [6]. Apart from germ cells, the testes also contain Sertoli cells (SCs), a decrease in SCs results in a proportional reduction in germ and Leydig cells, ultimately impacting fertility and overall health [7]. The quantity and quality of SCs are crucial for maintaining testicular homeostasis. A reduction in the number of SCs leads to a proportional decrease in the numbers of both germ and Leydig cells, which subsequently affects fertility and health [8]. Earlier research has demonstrated that HS reduces the number of SCs and decreases spermatogenesis [9]. However, the understanding of HS-induced damage to testicular SCs remains insufficient. The potential mechanisms linking HS and SCs apoptosis are unclear and require further investigation.

Mitochondria play a critical role in male reproduction. These organelles generate most of the ATP required for the growth, proliferation, and metabolism of SCs [10, 11]. Mitochondria, as the primary energy-generating organelles in the cell, are crucial for maintaining cellular function. The quality control of mitochondria depends on their dynamic balance [12]. Mitochondrial dynamics, such as fission and fusion, regulate the continuous cycling of mitochondria. Mitofusin 1 (MFN1), Mitofusin 2 (MFN2), and optic atrophy 1 (OPA1) are key proteins that regulate mitochondrial fusion, ensuring proper function and structure of mitochondria. Mitochondrial fission, in contrast, is regulated by fission protein 1 (FIS1) and dynamin-related protein 1 (DRP1), which facilitate mitochondrial cleavage and division. Maintaining a proper equilibrium between mitochondrial fusion and fission is vital for supporting cellular function and overall health [13]. These processes are closely related to mitochondrial energy metabolism, calcium homeostasis, redox balance, mitophagy and cell fate [14]. Mitophagy is a lysosomal process responsible for recognizing, eliminating, and recycling dysfunctional mitochondria [15]. Mitophagy occurs in two main stages: the early stage involves isolating damaged mitochondria into isolation membranes (autophagy initiation). The late stage is responsible for degrading the isolated components and depends on fully functional autophagosomes and lysosomes [14]. Currently, how HS influences intracellular stress signals and how these influence mitochondrial activities at the molecular level remains a challenging issue, requiring further research.

Melatonin, a hormone produced by the pineal gland, plays a crucial role in regulating various biological functions. In addition to its role in regulating the circadian rhythm, melatonin acts as a potent antioxidant [16]. Melatonin, an evolutionarily conserved metabolite derived from tryptophan, undergoes synthesis, transport, and metabolism in the mitochondria [17]. It has been found that melatonin targets SCs and can prevent oxidative stress in immature SCs [18]. While it is known that melatonin can mitigate HS-induced apoptosis in germ cells, the mechanism by which melatonin rescues SCs from apoptosis by regulating mitochondrial homeostasis remains unclear.

In this study, HS model was established in male goats in vivo. Primary goat SCs were exposed to HS in vitro. The results indicated that HS exposure caused defects in goat testicular tissue, decreased sperm quality, and induced apoptosis in SCs. Mechanistically, HS-induced mitochondrial-dependent apoptosis in SCs is likely due to excessive mitochondrial fission and inhibition of autophagic flux mediated by ROS-DRP1. The classical antioxidant melatonin protected SCs from HS-induced mitochondrial-dependent apoptosis by maintaining mitochondrial homeostasis. These findings provide deeper insights into the molecular mechanisms through which heat stress disrupts mitochondrial homeostasis in goat SCs. They also provide a basis for targeting mitochondrial homeostasis to mitigate the harmful effects of HS on male goat reproduction.

## Materials and methods

### Ethics statement

The animal experiments strictly followed the Chinese guidelines for animal research and were approved by the Institutional Animal Care and Use Committee at Northwest A&F University.

### Chemicals and reagents

Unless otherwise specified, all reagents and chemicals were sourced from Cell Signaling Technology (Danvers, MA, USA).

### Animals

In this study, the goats were Guanzhong dairy goats from Aonike Guanzhong Dairy Goat breeding farm, Fuping, Shaanxi province, China. Six male dairy goats, aged approximately 1.0 ± 0.3 years, with a weight of 45.0 ± 1.2 kg (Control) and 46 ± 0.5 kg (HS) were selected and assigned to control group (*n* = 3) and HS group (*n* = 3). The goats were proven breeders with a history of fertility and were free from any internal or reproductive health issues. It was ensured that the goats had sufficient drinking water and feed. Additionally, they had free access to water, feed, and mineral licks at all times. This study was conducted during the summer months (July–August). All goats were housed in sheds. In the HS group, goats were placed outdoors on an open exercise yard for 6 to 9 h per day (either from 10:00 to 16:00 or from 09:00 to 18:00) for a duration of 7 weeks, while ensuring that they had free access to feed and water outside the sheds.

### Measurement of environmental variables

Temperature and relative humidity were measured daily at the experimental site with a wet bulb thermometer (Zeal, Merton, London, UK) to measure the environmental variables. These measurements were used to calculate the temperature-humidity index (THI = [0.8 × air temperature (°C)] + [(% relative humidity/100) × (air temperature − 14.4)] + 46.4), as previously described [19]. The THI, derived from the dry bulb temperature (Tdb) and relative humidity (RH), represents an indicator for assessing the levels of HS experienced by the goats. Specifically, a THI value below 72 denotes no HS, values in the range of 73–77 indicate mild HS, 78–89 represents moderate HS, and values exceeding 90 indicate severe HS. During the entire experimental period, the morning THI values inside and outside the sheds were 68.7 ± 0.13 and 75.5 ± 0.2, respectively, while the afternoon THI values were 72.9 ± 0.32 and 81.5 ± 0.46, respectively. The THI indicated that the animals inside the sheds were not under stress, and the difference in THI between inside and outside the sheds was highly significant (*P* < 0.01).

### Semen collection and analysis

Semen was collected from the goats using the artificial vagina method and was immediately transferred to the laboratory. Two weeks prior to the start of the study, semen analysis was performed to ensure no significant differences in sperm quality among the goats. Semen was collected at 8:00 every 7 d after the start of the experiment. For consistency in subsequent analyses, the semen was diluted with a commercial Modina extender. The sperm quality and antioxidant enzyme levels were measured in all semen samples. Sperm quality was evaluated in vitro using a Computer-Assisted Sperm Analysis (CASA) system. SYBR-14/PI (LIVE/DEAD® Sperm Motility Kit) and Fluorescein isothiocyanate-labeled peanut agglutinin (FITC-PNA, Thermo Fisher, Waltham, MA, USA) was employed to assess sperm membrane and acrosome integrity. Each test was performed in triplicate.

### Testis collection

Goat testes were harvested for subsequent histological examination. The goats were first anesthetized with sodium pentobarbital. A sterile scalpel was then used to make an incision in the scrotum and the testes were removed. One testis was weighed and fixed in 4% paraformaldehyde for 24 h to conduct histological and immunohistochemical analyses. The other testis was promptly frozen in liquid nitrogen and stored at −80 °C for subsequent protein extraction.

### Hematoxylin and eosin (HE) staining, immunohistochemistry, and TUNEL assays

After deparaffinization, the sections (5 μm) were rehydrated using xylene and an ethanol gradient. After staining with HE, the morphology of the tissues was evaluated. Immunohistochemistry was conducted following previously described method [20]. SOX9 and WT1 staining was used to identify SCs, and SOX9/WT1-positive cells were counted in 25 randomly selected fields per tissue section. Apoptosis was assessed using a one-step TUNEL apoptosis detection kit (Meilunbio, Shanghai, China). To further confirm apoptosis in SCs, the tissue sections were blocked with 10% BSA and then incubated with SOX9 (1:50) at 37 °C for an additional 2 h following TUNEL staining. Subsequently, the sections were treated with Cy3–conjugated goat anti-mouse antibody (1:1,000) at 37 °C for 1 h and analyzed using laser confocal microscopy (Olympus, Tokyo, Japan).

### Transmission electron microscopy of testicular tissue

Testicular tissues were first fixed in 3% glutaraldehyde and subsequently treated with 1% osmium tetroxide. After dehydration with graded acetone solutions, the samples were infiltrated and embedded in Epon 812 resin. Semi-thin sections were stained with methylene blue, while ultra-thin sections were cut using a diamond knife and stained with uranyl acetate and lead citrate. The prepared sections were examined under transmission electron microscopy (TEM) using a JEM-1400-FLASH microscope (JEOL, Tokyo, Japan).

### Cell culture and heat stress treatment

The testicular tissues were carefully removed from the tunica albuginea and cut into 1–3 mm^3^ fragments using scissors. These fragments were digested with hyaluronidase and collagenase IV at 37 °C for 20 min. The resulting suspension was passed through a 40-μm cell strainer to isolate the cells. Following centrifugation at 1,200 r/min for 5 min, the cells were resuspended in DMEM/F12 medium supplemented with 10% FBS and then plated in cell culture dishes. The SCs adhered to the culture dish surfaces within 6 h. The extraction of primary goat SCs followed the previously established protocol [20], using specific markers (SOX9, WT1), with a purity of ≥ 95% required for subsequent experiments. HS was induced by incubating SCs at 42 °C for 30 min, with cells maintained at 36 °C serving as controls. Additionally, SCs were exposed to melatonin (MCE, Shanghai, China) for 24 h. For the combined treatment, cells were pre-treated with melatonin for 24 h before undergoing the 30 min HS.

### ATP levels

Goat SCs were plated at a density of 1.0 × 10^5^ cells per well in 6-well plates and incubated overnight. Following HS treatment, the cells were harvested and lysed for ATP measurement. ATP levels were assessed using a commercial ATP Assay Kit (Beyotime, Shanghai, China) and detected with a multifunction microplate reader (PerkinElmer, Wal-tham, MA, USA).

### Cell counting kit-8 (CCK-8) assay

Goat SCs were plated in 96-well plates at a density of 1 × 10^3^ cells per well and incubated overnight. After heat stress treatment, cell viability was evaluated using the CCK-8 assay, following the manufacturer’s protocol. Absorbance at 450 nm was recorded using a microplate reader (Bio-Tek, Everett, WA, USA), and cell viability was determined according to the provided formula.

### Measurement of mitochondrial calcium

Mitochondrial Ca^2+^ levels in SCs were measured using the fluorescent dye Rhod-2 AM (Solarbio, Beijing, China). The SCs were first incubated in HBSS buffer (lacking Ca^2+^ and Mg^2+^) with 4 µmol/L Rhod-2 AM at 37 °C for 30 min in the dark. Following this, the SCs were washed and incubated in fresh HBSS buffer for an additional 30 min. Confocal microscopy (Olympus, Tokyo, Japan) was then used to capture cellular images.

### Detection of apoptotic cells

The apoptosis of goat SCs was assessed using Annexin V and propidium iodide (PI) staining (Beyotime, Shanghai, China). A total of 1 × 10^5^ cells were suspended in 195 μL of binding buffer, combined with 5 mL Annexin V-FITC and 10 μL PI, and incubated for 30 min in the dark. The proportion of apoptotic cells was determined via flow cytometry.

### Measurement of mitochondrial ROS

Mitochondrial superoxide generation was analyzed using MitoSOX Red (Solarbio, Beijing, China). The cells were exposed to MitoSOX Red in darkness for 30 min. Following incubation, ROS in the mitochondria was quantified using a multifunction microplate reader (PerkinElmer, Waltham, MA, USA).

### Measurement of mitochondrial membrane potential (MMP)

The MMP was determined using the JC-1 Mitochondrial Membrane Potential Assay Kit (Beyotime, Shanghai, China). After collection, the cells were resuspended in 0.5 mL of JC-1 working solution at a density of 2 × 10^5^ cells per sample and incubated at 37 °C for 20 min. Flow cytometry was used to measure MMP, and the data were analyzed using Flow J software.

### Mitochondrial staining and morphological analysis

Goat SCs were plated in confocal dishes at a density of 1.0 × 10^5^ cells per well and cultured overnight. Following HS treatment, Mito-Tracker Red CMXRos (Beyotime, Shanghai, China) was added, and the cells were incubated with the dye at 37 °C for 30 min in darkness. To differentiate the mitochondria from the background, a threshold was applied to the images using the method of Yi et al. [21], followed by particle analysis. The aspect ratio and form factor, reflecting mitochondrial length and branching, respectively, were calculated for individual cells. For each treatment, 20–30 cells (analyzing over 500 mitochondria) were examined. Mitochondria with tubular or filamentous structures were classified as normal, while those with a punctate appearance were considered damaged. The percentage of cells showing these mitochondrial forms was calculated from at least 100 cells per treatment. This analysis allowed for a thorough evaluation of mitochondrial structure and health under different experimental conditions.

### Immunofluorescence

SCs were stained for immunofluorescence as detailed in previous protocols [20]. After HS exposure, the cells were fixed in paraformaldehyde (4%) and incubated with primary antibodies overnight. Following PBS washes, the appropriate secondary antibodies were added and incubated at 37 °C for 1 h. Images were obtained with a confocal microscope (Olympus, Tokyo, Japan), and fluorescence intensity was analyzed and quantified using ImageJ software.

### Western blotting

Western blotting was performed as previously described [20], with modifications to the antibodies used. Details of the primary antibodies used in the present study are provided in Supplementary Table S1. After overnight incubation with the primary antibodies, the samples were treated with the corresponding secondary antibodies. Blots were visualized using enhanced chemiluminescent reagents. Gray value analysis and quantification were conducted using ImageJ software.

### Data analysis

In this study, all parameters were measured with three biological replicates. Data were obtained from three independent experiments and are expressed as mean ± SD. GraphPad Prism 8.0 was utilized for statistical analysis and visualization. Two groups were analyzed using Student’s *t*-test. Differences in mean values among multiple groups were evaluated using one-way ANOVA followed by Bonferroni’s multiple comparisons test. Statistical significance was set at *P* < 0.05.

## Results

### HS leads to a decline in goat semen quality

**Fig. 1:**
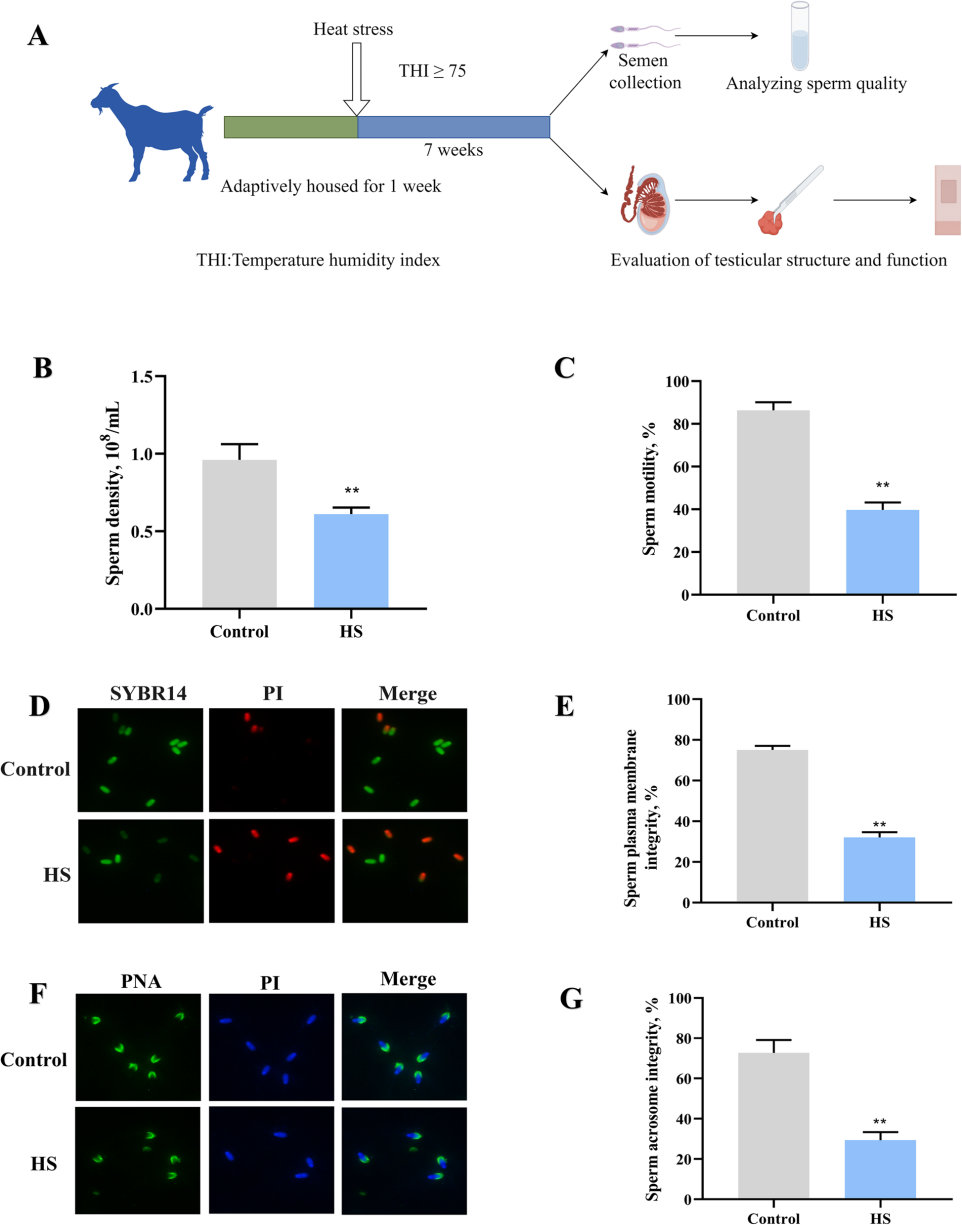
HS leads to a decline in sperm quality. **A** Experimental process showing the effects of HS on goat testis SCs. **B** Sperm density. **C** Sperm motility. **D** Sperm acrosome integrity. **E** Quantification of sperm acrosome integrity. **F** Sperm plasma membrane integrity. **G** Quantification of sperm plasma membrane integrity. *P < 0.05, **P < 0.01 vs. control

Semen was collected to detect semen quality from goats after HS exposure. The results are illustrated in Fig. 1. In comparison to the control group, HS significantly reduced the sperm count and density (*P* < 0.01) (Fig. 1B and C). Further testing of sperm quality revealed that the integrity of the sperm plasma membrane (Fig. 1D and E) and acrosome (Fig. 1F and G) in HS group exhibited a significant reduction compared to the control group (*P* < 0.01). These results indicate that HS reduces the quality of goat semen, thereby affecting reproductive capability.

### HS-induced testicular damage and a reduction in the number of SCs

**Fig. 2:**
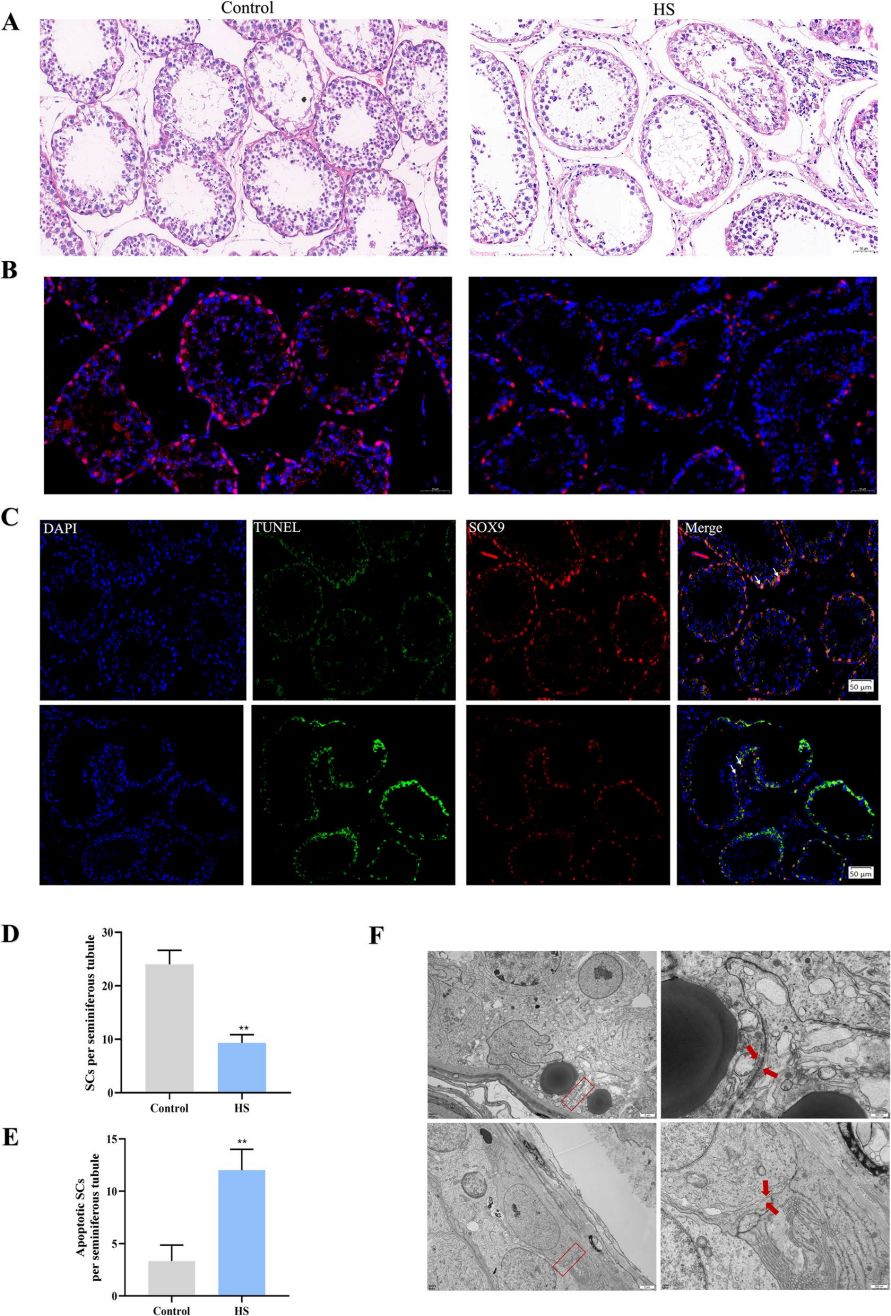
HS reduces SC numbers and induces apoptosis in the testis. **A** Representative HE staining of testis tissue viewed under light microscopy (scale bar: 50 μm). **B** Representative image of testicular immunofluorescence with SOX9 antibody (scale bar: 50 μm). **C** Immunohistochemistry of testis tissue combined with TUNEL staining for in vivo analysis of Sertoli cell apoptosis. **D** Quantification of SOX9-positive cells in 25 randomly selected seminiferous tubules per group (white arrows indicating SOX9 and TUNEL double-positive cells). **E** Quantification of SOX9 and TUNEL double-positive cells in 25 randomly selected seminiferous tubules per group. **F** Transmission electron microscopy images of changes in tight junctions between SCs. *P < 0.05, **P < 0.01 vs. control

To elucidate the effects of HS on goat testes at the tissue level, morphological analysis of the testes was conducted using HE staining. The HE results demonstrated that the spermatogenic tubules in the testes of goats from the control group had an intact lumen structure and the germ cells were densely packed and distinctly visible. However, HE-stained sections in HS groups showed that heat exposure resulted in structural damage to the testes, disrupting the architecture of the seminiferous tubules, disorganizing the cell layers, and reducing the number of layers (Fig. 2A). Immunofluorescence staining of testicular tissue using the SCs-specific marker SOX9 showed that HS exposure significantly decreased the number of SCs in goat testes compared to the control group (Fig. 2B and D). To assess whether HS induces apoptosis in SCs in vivo, a combination of TUNEL assay and immunohistochemistry was utilized. The findings revealed a marked increase in TUNEL-positive SCs in goat testes exposed to HS (Fig. 2C). Moreover, quantification confirmed that HS exposure resulted in a significant reduction in SC numbers and a pronounced rise in TUNEL-positive SCs (Fig. 2E). SCs play a critical role in maintaining the tight junctions of the testes. TEM analysis further confirmed that HS disrupted the tight junctions between adjacent SCs (Fig. 2F).

### HS-induced apoptosis and ROS production in primary goat SCs

**Fig. 3:**
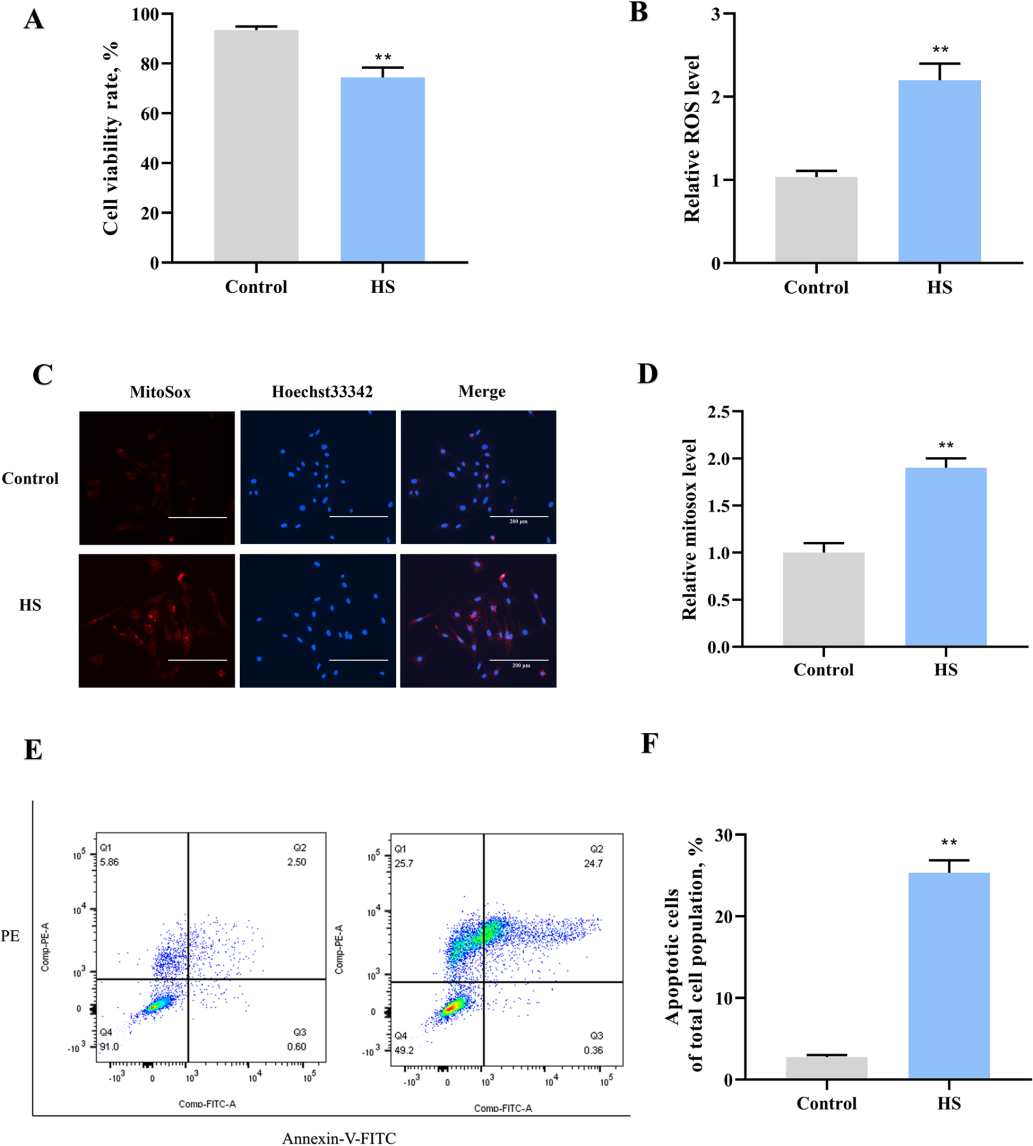
HS induces a decrease in cell viability and an increase in ROS and apoptosis in SCs. **A** SCs were exposed to 43 °C for 30 min. Cell viability was assessed by the CCK-8 assay kit. **B** ROS levels in SCs after HS exposure. **C** and **D** Representative images and assessment of mitochondrial ROS were conducted with the mitochondrial ROS kit. **E** and **F** Flow cytometry analysis of cell apoptosis in SCs after exposure to 43 °C for 30 min (Q1: mechanical trauma; Q2: late apoptosis; Q3: early apoptosis; Q4: living cells). *P < 0.05, **P < 0.01 vs. control

Primary goat SCs were isolated (Fig. S1), and an in vitro HS model was established to investigate the mechanism behind HS-induced apoptosis in SCs. The results indicated that SCs viability significantly decreased after HS exposure compared to the control group (*P* < 0.01) (Fig. 3A). We then assessed ROS levels and apoptosis in SCs after HS treatment. HS exposure caused an increase in ROS levels (Fig. 3B) and mitochondrial ROS (Fig. 3C and D) in SCs. Furthermore, HS exposure also significantly increased apoptosis in SCs. These results suggest a link between HS and apoptosis in goat SCs.

### HS induced excessive mitochondrial fission in goat SCs

**Fig. 4:**
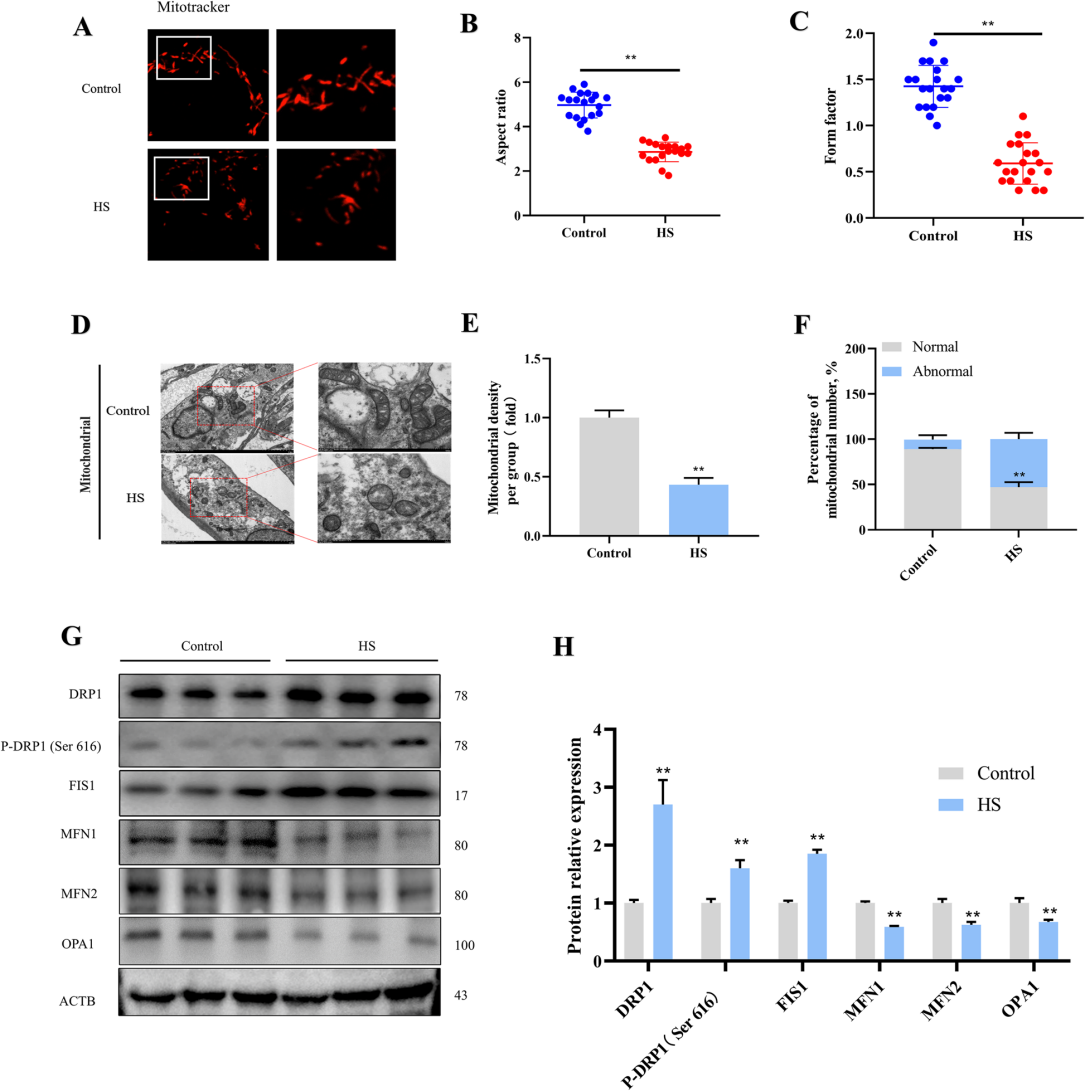
HS leads to mitochondrial fragmentation in SCs. **A** Mitochondria in SCs exposed to HS were labeled using Mito-Tracker Red CMXRos. **B** Morphological parameters, including the aspect ratio, were evaluated. **C** Morphology factors (shape factor) were evaluated. **D** Transmission electron microscopy was used to assess the effects of HS on the number and structure of mitochondria in SCs. **E** Mitochondrial density was quantified. **F** Quantitative analysis examined normal and abnormal mitochondrial morphology in SCs after HS exposure. **G** Expression levels of proteins related to mitochondrial dynamics in SCs. **H** Quantification of mitochondrial dynamics-related proteins using Image J. *P < 0.05, **P < 0.01 vs. control

To clarify the impact of HS on mitochondrial structure in SCs, we stained the mitochondria using mitotracker red dye. The mitochondria of cells in the control group exhibited tubular or filamentous structures that formed interconnected networks. Conversely, mitochondria in the HS group appeared punctate, with fewer interconnections, indicative of mitochondrial fragmentation (Fig. 4A). Quantitative assessment of mitochondrial length (aspect ratio) and branching (form factor) verified that HS exposure led to mitochondrial fragmentation in SCs (Fig. 4B and C). Furthermore, the mitochondrial ultrastructure was analyzed using TEM. TEM showed that HS caused mitochondrial swelling, disrupted the structure of the cristae, and increased mitophagy in SCs (Fig. 4D–F). Subsequently, mitochondria were then isolated from SCs to evaluate the expression of proteins involved in mitochondrial fission and fusion. As depicted in Fig. 4G and H, HS treatment elevated the expression of DRP1, P-DRP1, and FIS1. Notably, following HS exposure, DRP1 relocated from the cytoplasm to the mitochondria in SCs. In contrast, HS reduced the levels of MFN1, MFN2, and OPA1 in SC mitochondria. These findings suggest that HS promotes DRP1 translocation to the mitochondria in SCs, leading to excessive mitochondrial fission.

### HS induced mitochondrial dysfunction and mitochondria-dependent apoptosis in goat SCs

**Fig. 5:**
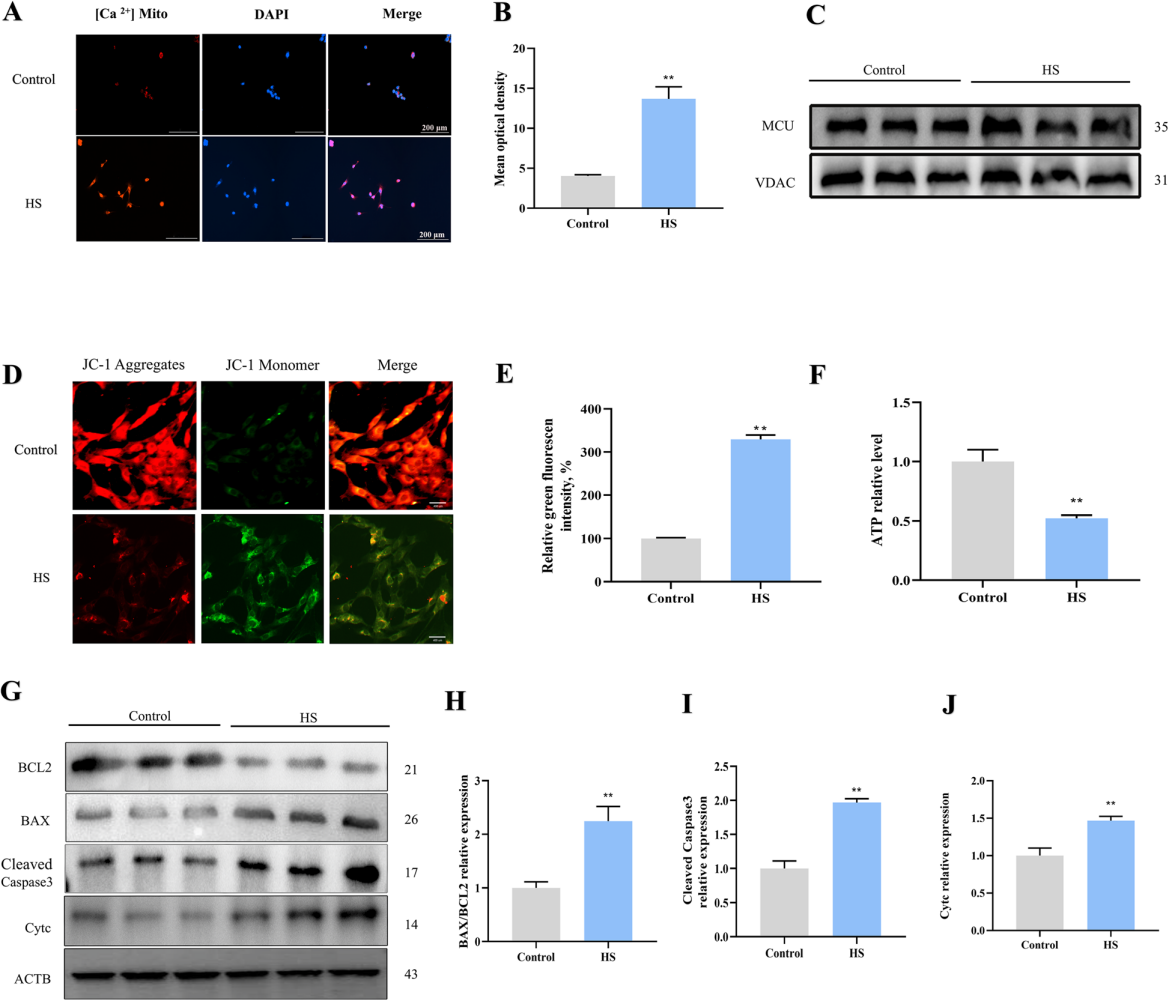
HS induces mitochondrial dysfunction and apoptosis in SCs. **A** and **B** Mitochondrial Ca^2+^ levels in SCs after HS exposure. **C** Protein expression levels of the MCU in SCs after HS exposure. **D** and **E** JC-1 staining was employed to assess the effect of HS on mitochondrial membrane potential. **F** ATP levels in SCs were measured using luciferase-based assays. **G** Expression of proteins involved in mitochondria-dependent apoptosis. **H**–**J** Quantification of proteins using Image J. *P < 0.05, **P < 0.01 vs. control

Ca^2+^ homeostasis is closely related to the regulation of mitochondrial dynamics and apoptosis. The current findings indicate that HS increased Ca^2+^ levels in mitochondria (Fig. 5A and B). Similarly, the expression of the MCU, a protein responsible for Ca^2+^ uptake, was also elevated after HS exposure (Fig. 5C). Additionally, mitochondrial function indicators, including MMP and ATP levels, were assessed to further clarify the impact of HS on mitochondrial function. Analysis of mitochondrial membrane potential revealed that HS increased the JC-1 ratio of monomers (green fluorescence) to aggregates (red fluorescence), indicating mitochondrial membrane disruption (Fig. 5D and E). Following HS exposure, ATP content in SCs decreased by 48% (Fig. 5F). Finally, mitochondrial apoptosis-related proteins were examined. HS exposure increased the level of BAX, Cleaved-caspase3, and Cytc compared to the control group (Fig. 5G–J), suggesting that HS-induced apoptosis may be due to Cytc activating the caspase cascade pathway.

### Inhibition of mitochondrial fission can block mitochondria-dependent apoptosis

**Fig. 6:**
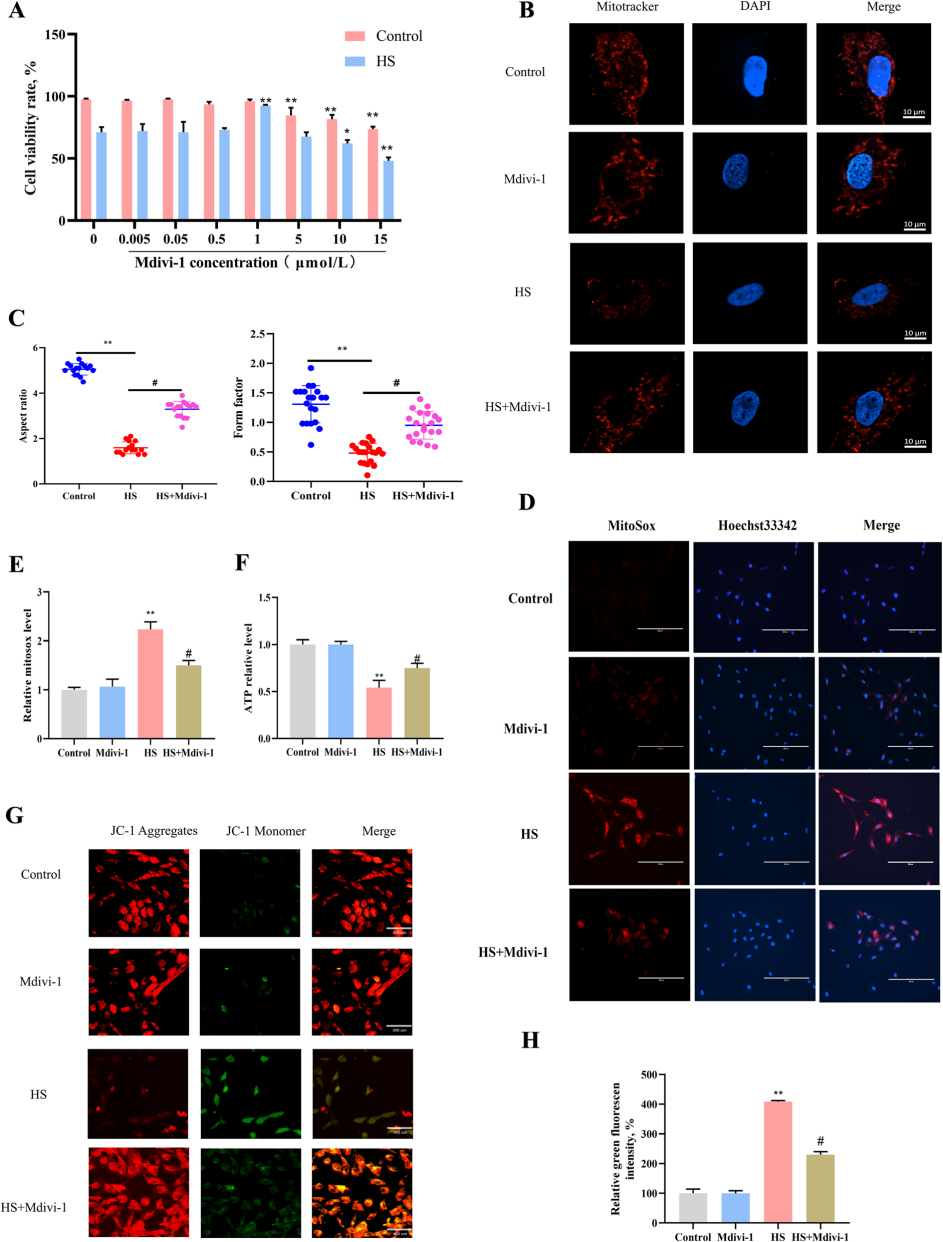
The DRP1 inhibitor Mdivi-1 ameliorates mitochondrial dysfunction. **A** SCs were treated with different concentrations of Mdivi-1, and cell viability was assessed using the CCK-8 assay. **B** Effects of Mdivi-1 pretreatment on mitochondrial morphology in SCs. **C** Morphological factors (aspect ratio and shape factor) were evaluated after Mdivi-1 pretreatment. **D** and **E** Representative images and quantitative analysis of mitochondrial ROS after Mdivi-1 pretreatment. **F** ATP content were measured in SCs following Mdivi-1 pretreatment. **G** and **H** Representative images and analysis of mitochondrial membrane potential (ΔΨm) after Mdivi-1 pretreatment. *P < 0.05, **P < 0.01 vs. control; ^#^*P* < 0.05 vs. HS

To determine whether HS-induced excessive mitochondrial fission leads to mitochondria-dependent apoptosis, the DRP1 inhibitor Mdivi-1 was used in primary goat SCs. CCK-8 results showed that 1 μmol/L Mdivi-1 had no significant effect on SCs viability in the absence of HS exposure (Fig. 6A); therefore, 1 μmol/L Mdivi-1 was used in subsequent experiments. Compared to the HS exposure group, Mdivi-1 reduced HS-induced mitochondrial fragmentation in SCs (Fig. 6B), while quantitative assessment of mitochondrial dimensions (aspect ratio) and network complexity (form factor) showed an increase (Fig. 6C). Interestingly, Mdivi-1 also reduced the HS-induced increase in mitochondrial ROS (Fig. 6D and E), and it rescued the HS-induced decrease in ATP levels (Fig. 6F) and MMP (Fig. 6G and H) in SCs.

**Fig. 7:**
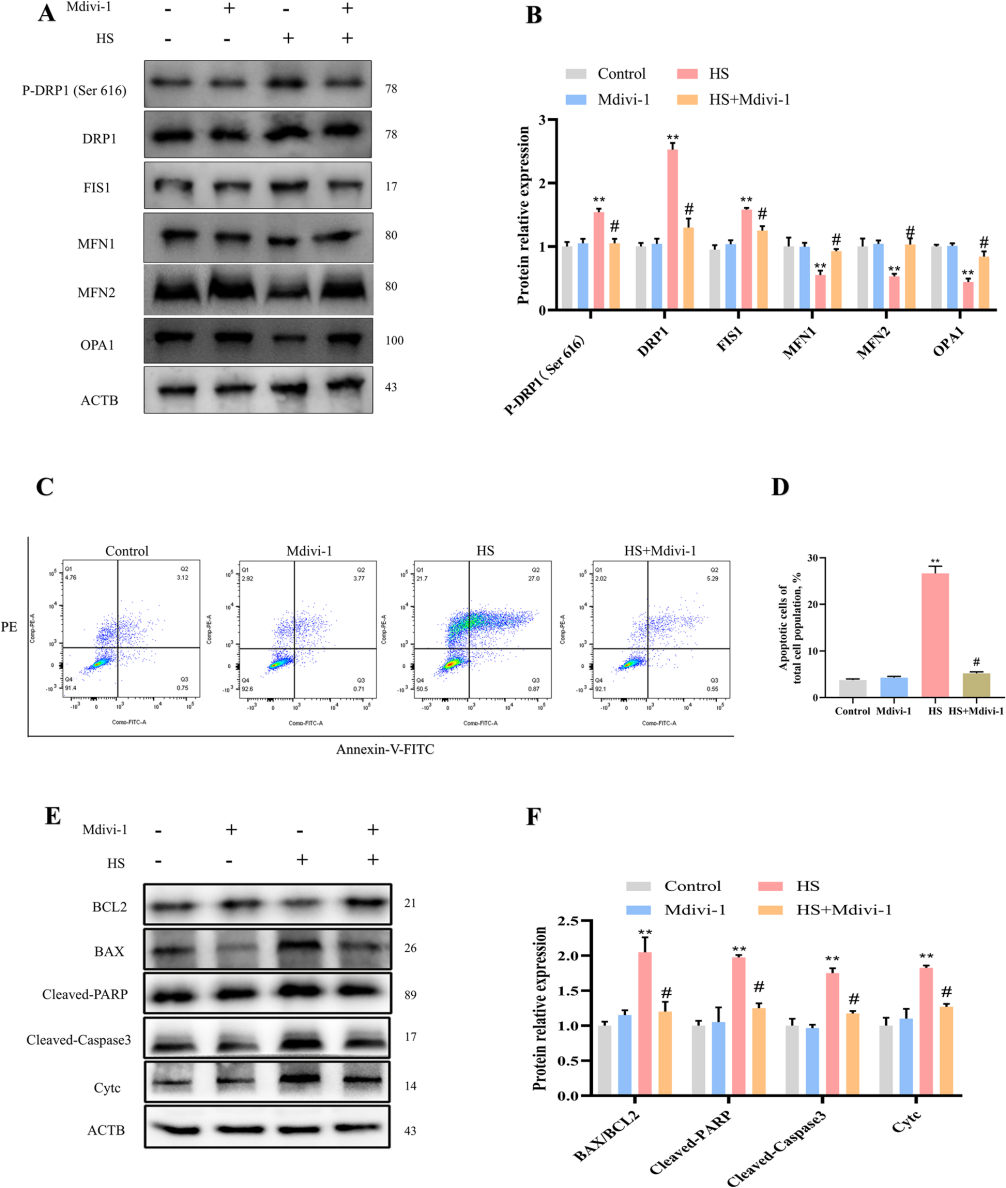
The DRP1 inhibitor Mdivi-1 suppresses excessive mitochondrial fission and mitochondria-dependent apoptosis. **A** The effects of Mdivi-1 on the expression of proteins associated with mitochondrial dynamics. **B** Quantification of mitochondrial-related protein expression. **C** and **D** Analysis of SC apoptosis using flow cytometry. **E** Effects of Mdivi-1 pretreatment on the expression of mitochondria-dependent apoptotic proteins. **F** Quantification of mitochondria-dependent apoptotic proteins. *P < 0.05, **P < 0.01 vs. control; ^#^ *P* < 0.05 vs. HS

To clarify the role of DRP1-mediated mitochondrial fission in SCs apoptosis, the expression levels of proteins related to mitochondrial dynamics and apoptosis were analyzed. Mdivi-1 reduced the expression of mitochondrial fission proteins (DRP1, P-DRP1, FIS1) and increased the expression of fusion proteins (MFN1, MFN2, OPA1) following HS exposure (Fig. 7A and B). Furthermore, flow cytometry analysis showed that Mdivi-1 reduced apoptosis (Fig. 7C and D). Mdivi-1 decreased the expression of mitochondria-dependent apoptosis-related proteins (Cleaved-PARP, BAX, Cleaved-caspase3, Cytc) compared to the HS group (Fig. 7E and F). These findings suggest that Mdivi-1 suppresses DRP1-mediated mitochondrial fission, rescuing HS-induced mitochondria-dependent apoptosis in SCs.

### HS-induced mitochondrial dysfunction in SCs is associated with mitophagy

**Fig. 8:**
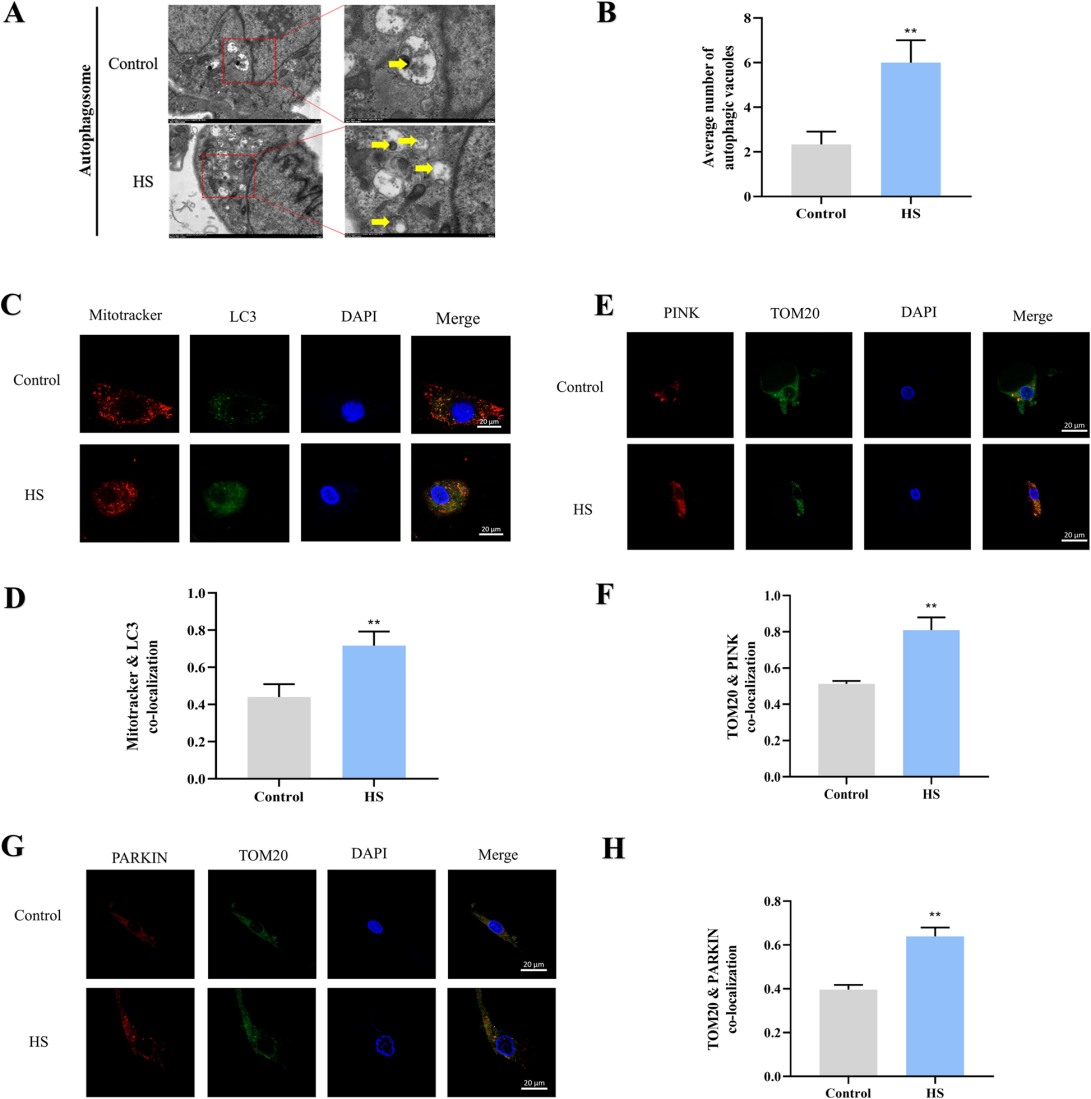
HS-induced mitochondrial fragmentation is associated with mitophagy. **A** Transmission electron microscopy images showing the effects of HS on mitophagy in SCs, with yellow arrows indicating autophagic vesicles. **B** Analysis and quantification of mitochondrial autophagic vesicles. **C** and **D** Exemplary confocal fluorescence images and related quantitative assessments showing co-localizations of mitotracker (red) and LC3 (green), scale bar = 10 μm; Blue: DAPI staining. **E** and **F** Exemplary confocal fluorescence images and related quantitative assessments showing co-localizations of TOM20 (green) and PINK (red), scale bar = 20 μm. Blue: DAPI staining. **G** and **H** Exemplary confocal fluorescence images and related quantitative assessments showing co-localizations of TOM20 (green) and PARKIN (red), scale bar = 20 μm. *P < 0.05, **P < 0.01 vs. control; ^#^ *P* < 0.05 vs. HS

Mitochondrial fission and mitophagy work together to maintain mitochondrial homeostasis. HS induced excessive mitochondrial fission and dysfunction, suggesting potential impairment of mitophagy. Therefore, we examined mitophagy-related markers. TEM results indicated that HS increased the number of autophagic vesicles (Fig. 8A and B). LC3 and mitotracker immunofluorescence showed strong colocalization in SCs after HS exposure (Fig. 8C and D). As expected, PINK (Fig. 8E and F), Parkin (Fig. 8G and H), and TOM20 immunofluorescence also showed strong colocalization compared to control group.

**Fig. 9:**
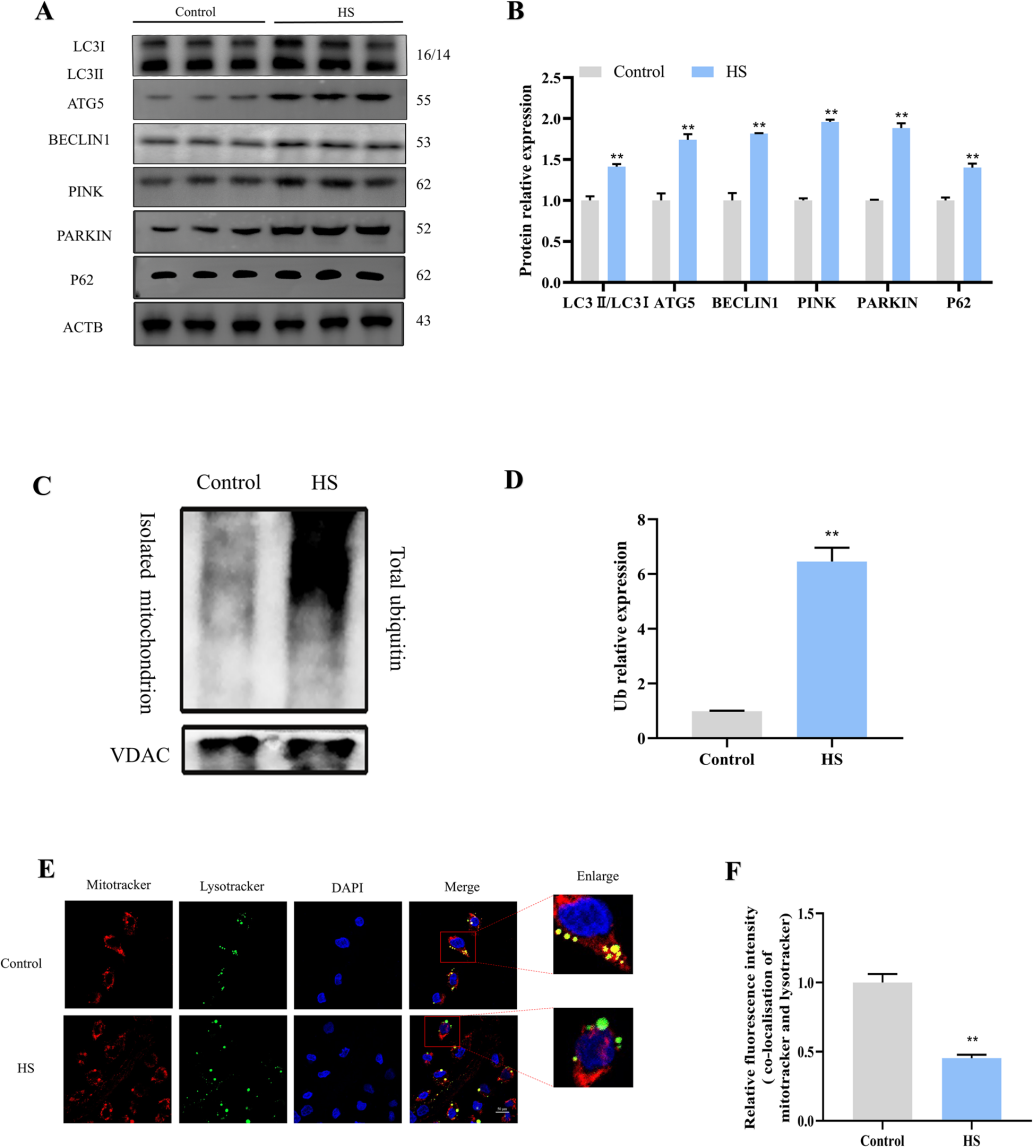
HS-induced mitochondrial fragmentation is associated with the inhibition of mitophagy flux. **A** and **B** Western blotting images and associated quantitative evaluations of autophagy markers in SCs. **C** and **D** Western blotting images and quantitative analyses of Ub expression in SCs after HS exposure. **E** and **F** Representative images and analysis of mitotracker (red) and lysotracker (green) colocalization in SCs using confocal microscopy. Scale bar = 20 μm. Blue: DAPI staining. *P < 0.05, **P < 0.01 vs. control; ^#^ *P* < 0.05 vs. HS

Additionally, the expression levels of mitophagy proteins LC3, Agt5, Beclin-1, PINK, and PARKIN were upregulated under HS conditions (Fig. 9A and B). PINK1-induced Parkin ubiquitination plays a role in the initiation of mitophagy, ubiquitination levels significantly increased after HS exposure (Fig. 9C and D). These findings suggest that HS triggers the initiation of mitophagy through excessive mitochondrial fission. Notably, the expression level of the mitophagy substrate P62 was elevated, indicating potential downstream impairment in mitophagy. Damaged mitochondria are typically engulfed by autophagosomes and transferred to lysosomes for degradation to maintain mitochondrial homeostasis. Further studies revealed that HS exposure significantly reduced the colocalization between mitochondria (mitotracker) and lysosomes (lyso tracker) (Fig. 9E and F), indicating that HS exposure hindered the fusion of autophagosomes with lysosomes, consequently disrupting mitophagy flux. Overall, these findings suggest that HS exposure initiates mitophagy while inhibiting mitophagy flux, leading to SC apoptosis.

### Melatonin rescues HS-induced mitochondria-dependent apoptosis in SCs by restoring mitochondrial homeostasis

**Fig. 10:**
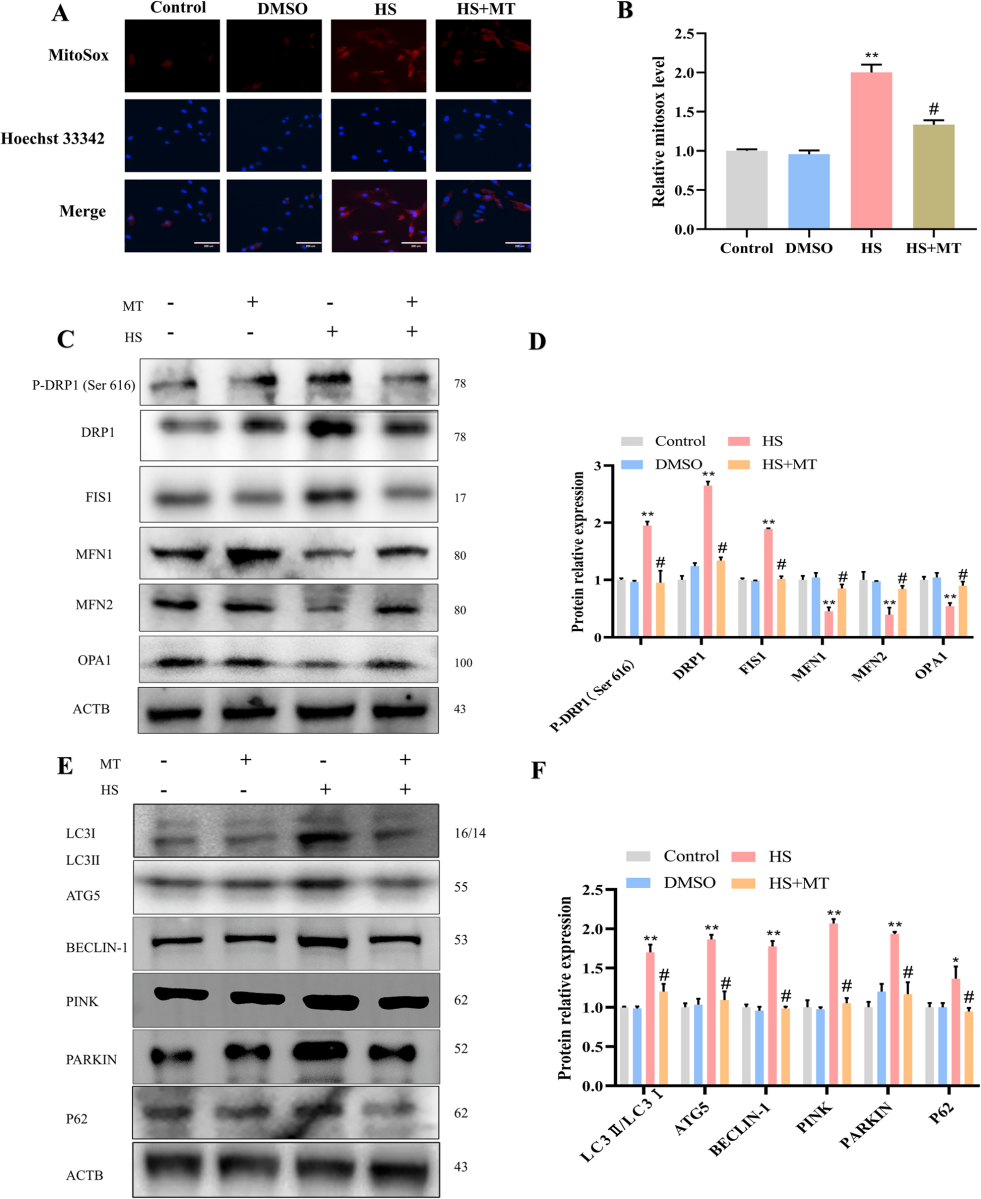
Melatonin rescues HS-induced mitochondrial homeostasis disruption in SCs. **A** and **B** Representative images and analysis of mitochondrial ROS in SCs after melatonin treatment. **C** The expression of proteins related to mitochondrial dynamics was analyzed using Western blot. **D** Quantification of mitochondrial dynamics-related proteins using ImageJ. **E** The expression of mitophagy-related proteins was examined through Western blot analysis. **F** Quantification of mitophagy-related proteins using ImageJ. *P < 0.05, **P < 0.01 vs. control; ^#^ *P* < 0.05 vs. HS

**Fig. 11:**
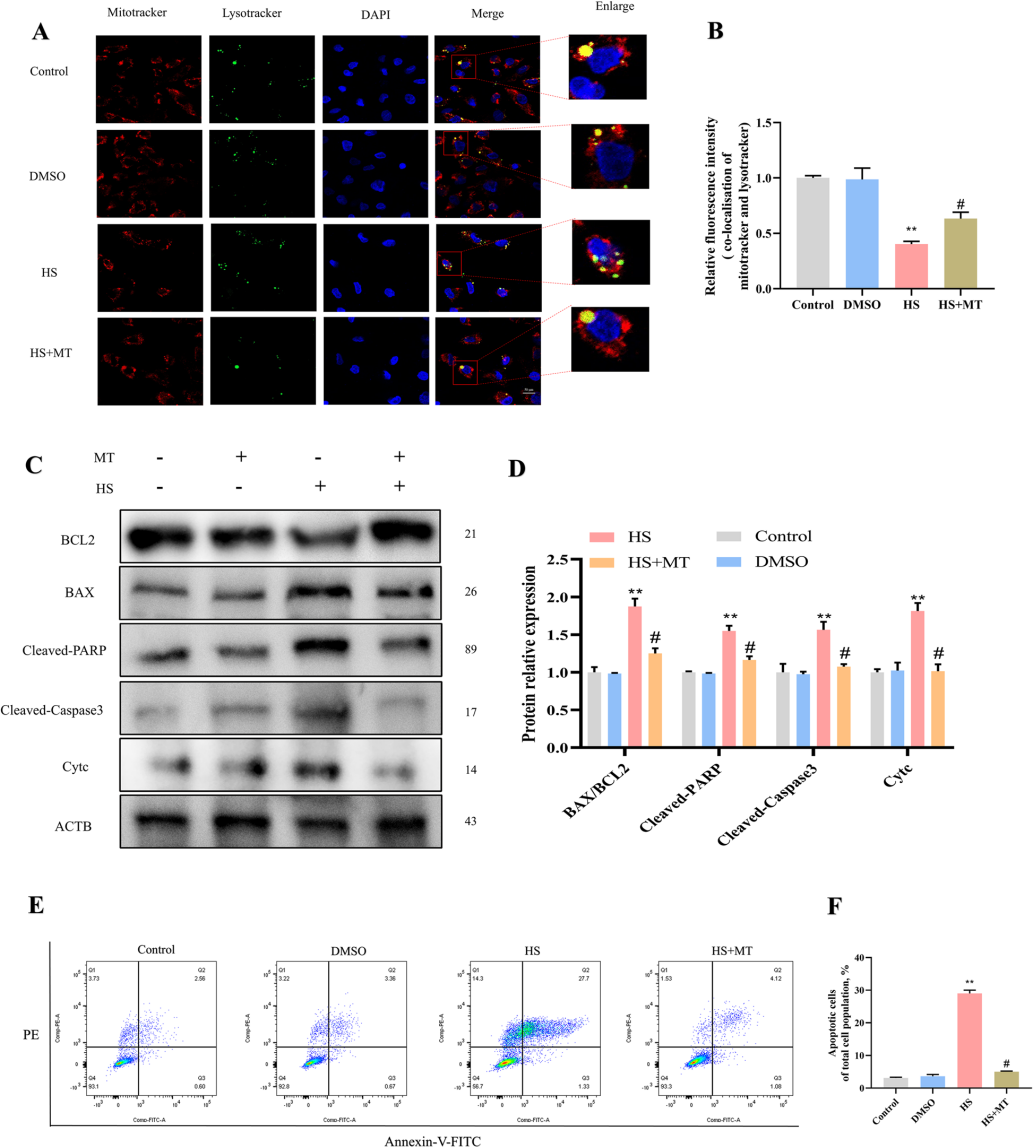
Melatonin inhibits mitochondria-dependent apoptosis. **A** and **B** Representative images and analysis of mitotracker (red) and lysotracker (green) colocalization in SCs using confocal microscopy. Scale bar = 50 μm. Blue: DAPI staining. **C** and **D** Western blotting images and quantitative analyses of Bax, cleaved-Caspase3, cleaved-PARP, and cytochrome c. **E** and **F** Cell apoptosis detected by flow cytometry. *P < 0.05, **P < 0.01 vs. control; ^#^ *P* < 0.05 vs. HS

To determine whether melatonin counteracts HS-induced apoptosis by maintaining mitochondrial homeostasis, we conducted a series of experiments. In this study, we found that lower concentrations of melatonin (0.005, 0.05, 0.5 μmol/L) maintained better SC viability compared to higher concentrations (1, 5, and 10 μmol/L). Moreover, melatonin at a concentration of 0.5 μmol/L significantly enhanced cell viability after heat stress compared to 0.05 μmol/L. Therefore, we selected 0.5 μmol/L melatonin for subsequent experiments in this study (Fig. S2). The findings demonstrated that melatonin supplementation mitigated the HS-induced rise in mitochondrial ROS levels (Fig. 10A and B). Additionally, our data indicated that melatonin inhibited the levels of proteins associated with mitochondrial fission while promoting the expression of mitochondrial fusion proteins in SCs following HS exposure (Fig. 10C and D). Further analysis of mitophagy-related proteins revealed that their expression was reduced (Fig. 10E and F), suggesting that melatonin inhibits rather than promotes mitophagy in SCs. Excitingly, the addition of melatonin downregulated the expression of P62, indicating that melatonin likely restored normal mitophagy flux. Next, we evaluated the impact of melatonin on mitophagy flux. In comparison to the HS group, melatonin significantly increased the colocalization between mitochondria and lysosomes (Fig. 11A and B), suggesting that melatonin restored the HS-induced blockade of mitophagy flux. Finally, we analyzed the expression of proteins related to mitochondria-dependent apoptosis. In comparison to the HS group, melatonin markedly decreased the levels of these proteins (Fig. 11C and D) and flow cytometry results also indicated that melatonin reduced HS-induced apoptosis (Fig. 11E and F). To further elucidate whether the inhibition of autophagic flux exacerbates SCs apoptosis following HS, we employed a mitophagy inhibitor (BafA1) and an activator (Rapa) to investigate SCs apoptosis after HS. Flow cytometry and Western blot analyses revealed that BafA_1_ significantly increased SCs apoptosis, whereas the mitophagy activator Rapa markedly attenuated HS–induced damage to SCs. BafA1 primarily inhibits mitophagy by blocking the fusion of autophagosomes with lysosomes, further demonstrating that HS suppresses autophagosome–lysosome fusion and impedes autophagic flux (Fig. S3). In a word, these findings suggest that melatonin rescues HS-induced mitochondria-dependent apoptosis by reducing mitochondrial ROS production and restoring mitochondrial homeostasis (including mitochondrial dynamics and mitophagy).

## Discussion

With global warming, livestock frequently experience HS during the summer. HS severely impacts the reproductive capability of goats. Previous studies had shown that HS disrupts the structure of testicular tissue, impairing spermatogenesis [22, 23]. SCs, which are crucial for coordinating spermatogenesis, regulate the differentiation of spermatogonia and maintain adult testis function. Prior research has indicated that HS can induce apoptosis in goat spermatogonia, adversely affecting spermatogenesis [24, 25]. Nevertheless, the underlying mechanisms about HS-induced apoptosis in SCs remain unclear. In this study, HS caused a decline in goat sperm quality, abnormal sperm morphology, and apoptosis of goat SCs. The decline in goat sperm quality and apoptosis of testicular SCs further validated the successful establishment of our in vivo heat stress model. Mechanistically, the apoptosis of SCs may result from excessive mitochondrial fission induced by ROS-DRP1 and the blockage of mitophagy flux.

Mitochondria are highly dynamic organelles responsible for regulating cellular energy metabolism and generating ATP [26]. The integrity of mitochondrial structure and function determines cell fate [27]. The generation of ROS results in oxidative damage to mitochondria. Interestingly, this oxidative damage in mitochondria results in the production of even more ROS, creating a vicious cycle [27]. In this study, we found that ROS levels in SCs significantly increased after exposure to HS. Additionally, HS caused an increase in mitochondrial fragmentation and the number of abnormally shaped mitochondria in SCs. Mitochondria continuously undergo fission and fusion to maintain their function and homeostasis [28]. Imbalanced mitochondrial fission and fusion can result in mitochondrial dysfunction [29]. In this study, HS exposure increased the expression of DRP1 and FIS1, while MFN1, MFN2, and OPA1 was decreased. Mitochondrial structure is closely linked to its functional performance. As expected, HS disrupted mitochondrial morphology, leading to mitochondrial dysfunction, which was evident by the rise in mitochondrial ROS, along with the reduction in mitochondrial membrane potential and ATP levels, observed following HS exposure in our study. Similar studies have also reported that excessive mitochondrial fragmentation cause mitochondrial dysfunction, leading to altered mitochondrial permeability, inhibition of the respiratory chain, and reduced ATP production [30–32]. Previous studies, along with our findings, indicated that HS leads to elevated mitochondrial ROS levels, which subsequently induces excessive mitochondrial fission and mitochondrial dysfunction. However, the exact mechanism by which HS causes mitochondrial fragmentation in SCs remains unclear and requires further investigation.

DRP1 plays a crucial role in driving mitochondrial fission, and under conditions of mitochondrial stress, DRP1 translocates from the cytoplasm to the mitochondria [13, 33]. DRP1 has been shown to oppose the activity of the anti-apoptotic protein BCL2 [34], while on the other hand, DRP1 enhances the activity of the pro-apoptotic protein BAX, inducing mitochondrial remodeling [35, 36]. DRP1 activity plays a role in altering mitochondrial membrane permeability and facilitating the release of cytochrome C. In this study, our results indicate that HS promotes the expression of DRP1 and activates cytochrome C release and the mitochondria-dependent apoptotic pathway. Furthermore, the DRP1 inhibitor Mdivi-1 was found to inhibit mitochondrial fission, thereby rescuing mitochondrial dysfunction, as evidenced by increased MMP, elevated ATP levels, reduced ROS levels, and decreased mitochondria-dependent apoptosis. Earlier research has revealed that DRP1-driven mitochondrial fission induces apoptosis in periodontal ligament stem cells [37]. Moreover, research has demonstrated that DRP1 recruitment to mitochondria induces ROS generation and mitochondrial fission, which is associated with mitochondrial dysfunction. After Mdivi-1 treatment, ROS generation and mitochondrial dysfunction were mitigated in neuronal cells [38]. Building on previous studies, it had been shown that inhibiting DRP1 can block mitochondrial fission and mitochondria-dependent apoptosis. Notably, the findings of this study revealed a significant rise in the expression of phosphorylated DRP1 at the Ser616 site in SCs. Previous studies had shown that phosphorylation of DRP1 at the Ser616 site can promote its expression [39]. Previous studies had shown that phosphorylation of DRP1 at the Ser616 site can promote its expression, which is consistent with our findings.

Calcium ions in mitochondria are involved in energy production, regulation of signal transduction, mitochondrial permeability transition, and even programmed cell apoptosis [40]. Under oxidative stress conditions, superoxide anions can disrupt calcium homeostasis within the endoplasmic reticulum and mitochondria, thereby triggering the activation of the intrinsic apoptotic pathway [41]. Additionally, an imbalance in calcium metabolism can lead to abnormal mitochondrial dynamics [42]. The mitochondrial calcium uniporter is a fully assembled complex consisting of the calcium-conducting channel (known as the MCU channel) and a series of regulatory proteins [43]. In this study, the Ca^2+^ content and the expression of MCU in SCs were significantly increased after HS exposure compared to the control group, indicating that HS exposure disrupted Ca^2+^ homeostasis. Based on previous findings, we hypothesize that HS likely alters Ca^2+^ homeostasis by increasing ROS levels, leading to abnormal mitochondrial dynamics. However, the specific molecular mechanisms require further investigation. Mitochondrial dynamics and mitophagy collaborate to ensure the maintenance of mitochondrial homeostasis. Studies have shown that after mitochondrial fission, autophagosomes engulf the fragmented mitochondria to facilitate mitophagy [28]. Recent evidence indicates that mitophagy has a dual role in responding to exogenous stress, both by protecting cellular homeostasis and inducing cell death through apoptosis [44, 45]. Studies have suggested that HS-induced autophagy may enhance SC function [46]. In this study, HS activated PINK-PARKIN-mediated mitophagy but hindered the fusion between autophagosomes and lysosomes, resulting in impaired mitophagy.

Melatonin is a classic mitochondria-targeted antioxidant [47]. Research has indicated that ROS in cells can, on one hand, promote mitophagy to maintain mitochondrial function, on the other hand, ROS suppress autophagy flux [48, 49]. In this study, HS induced excessive ROS production in SCs, which led to increased DRP1 expression. Notably, ROS also promoted DRP1 phosphorylation at the Ser616 site, further enhancing DRP1 expression. The increase in DRP1 leads to excessive mitochondrial fission. Additionally, DRP1 is essential for PINK1/PARKIN-mediated mitophagy, as it facilitates complex formation and directs it to sites of mitochondrial damage [50]. Subsequent studies revealed that HS initiated PINK1/Parkin-mediated mitophagy but inhibited the fusion of autophagosomes and lysosomes, resulting in impaired mitophagy. This defective mitophagy caused the accumulation of fragmented mitochondria and mitochondrial peroxides, ultimately exacerbating intrinsic apoptosis. Melatonin supplementation reduced ROS levels in SCs, which subsequently decreased the expression of mitochondrial fission proteins and increased the expression of mitochondrial fusion proteins. The reduction in DRP1 levels also led to decreased autophagy activity. Further investigation demonstrated that melatonin inhibited mitophagy, restored autophagic flux, and reduced mitochondria-dependent apoptosis.

## Conclusion

In summary, this study indicates that HS exposure induces excessive mitochondrial fission and mitochondrial dysfunction (increased ROS production, decreased ATP, and MMP levels), leading to mitochondria-dependent apoptosis in goat SCs. The blockade of mitophagy flux induced by HS further exacerbates mitochondria-dependent apoptosis. Melatonin reduces mitochondria-dependent apoptosis by scavenging ROS, maintaining mitochondrial homeostasis (by inhibiting excessive mitochondrial fission and restoring mitophagy flux).

## Supplementary Information

Additional file 1: Table S1. Detailed antibody information. Fig. S1. Isolation and characterization of primary SCs in goats. Fig. S2. Effect of different concentrations of melatonin treated for 24 h on cell viability. Fig. S3. Effects of inhibiting or promoting mitophagy on HS-induced apoptosis in SCs.

## Data Availability

All data generated or analyzed during this study are included.

## Abbreviations

ATP: Adenosine triphosphate
CASA: Computer-assisted sperm analysis
CCK-8: Cell counting kit-8
DRP1: Dynamin-related protein 1
FBS: Fetal bovine serum
FIS1: Fission protein 1
H&E: Hematoxylin and eosin
HS: Heat stress
MMP: Mitochondrial membrane potential
MFN1: Mitofusin 1
MFN2: Mitofusin 2
OPA1: Optic atrophy 1
PI: Propidium iodide
ROS: Reactive oxygen species
SC: Sertoli cell
TEM: Transmission electron microscopy
