# Supplementary material for: ROS-DRP1-mediated excessive mitochondrial fission and autophagic flux inhibition contribute to heat stress-induced apoptosis in goat Sertoli cells
Source: J Anim Sci Biotechnol. 2025 Apr 16;16:58. doi: 10.1186/s40104-025-01180-2 (PMC12001645; doi:10.1186/s40104-025-01180-2)
Supplement: Supplementary file 1 — Additional file 1: Table S1. Detailed antibody information. Fig. S1. Isolation and characterization of primary SCs in goats. Fig. S2. Effect of different concentrations of melatonin treated for 24 h on cell viability. Fig. S3. Effects of inhibiting or promoting mitophagy on HS-induced apoptosis in SCs. [file 40104_2025_1180_MOESM1_ESM.docx]

**Supplementary materials**

**Additional file 1**

**Table S1** Detailed antibody information

| **Type** | **Antibody name** | **Host/clonality** | **Manufacturer** | **Dilution ratio** |
| --- | --- | --- | --- | --- |
| Primary  antibodies | SOX9 | Mouse/Polyclonal | Proteintech | 1:800 |
|  | LC3 | Rabbit/Polyclonal | Bioss | 1:800 |
|  | TOM20 | Rabbit/Polyclonal | Proteintech | 1:800 |
|  | Ub | Rabbit/Polyclonal | Bioss | 1:800 |
|  | PARKIN | Rabbit/Polyclonal | Bioss | 1:800 |
|  | ATG5 | Mouse/Polyclonal | Bioss | 1:800 |
|  | BECLIN-1 | Rabbit/Polyclonaly | Proteintech | 1:800 |
|  | PINK1 | Rabbit/Polyclonal | Bioss | 1:800 |
|  | P62 | Rabbit/Polyclonal | Proteintech | 1:800 |
|  | β-actin | Rabbit/Polyclonal | Proteintech | 1:800 |
|  | MFN1 | Rabbit/Polyclonal | Proteintech | 1:800 |
|  | MFN2 | Mouse/Polyclonal | Bioss | 1:800 |
|  | OPA1 | Mouse/Polyclonal | Bioss | 1:800 |
|  | DRP1 | Rabbit/Polyclonal | Proteintech | 1:800 |
|  | P-DRP1 | Rabbit/Polyclonal | Proteintech | 1:800 |
|  | BAX | Rabbit/Polyclonal | Proteintech | 1:800 |
|  | BCL2 | Rabbit/Polyclonal | Proteintech | 1:800 |
|  | Caspase3 | Mouse/Polyclonal | Bioss | 1:800 |
|  | PARP | Mouse/Polyclonal | Bioss | 1:800 |
|  | Cytc | Rabbit/Polyclonal | Bioss | 1:800 |

**
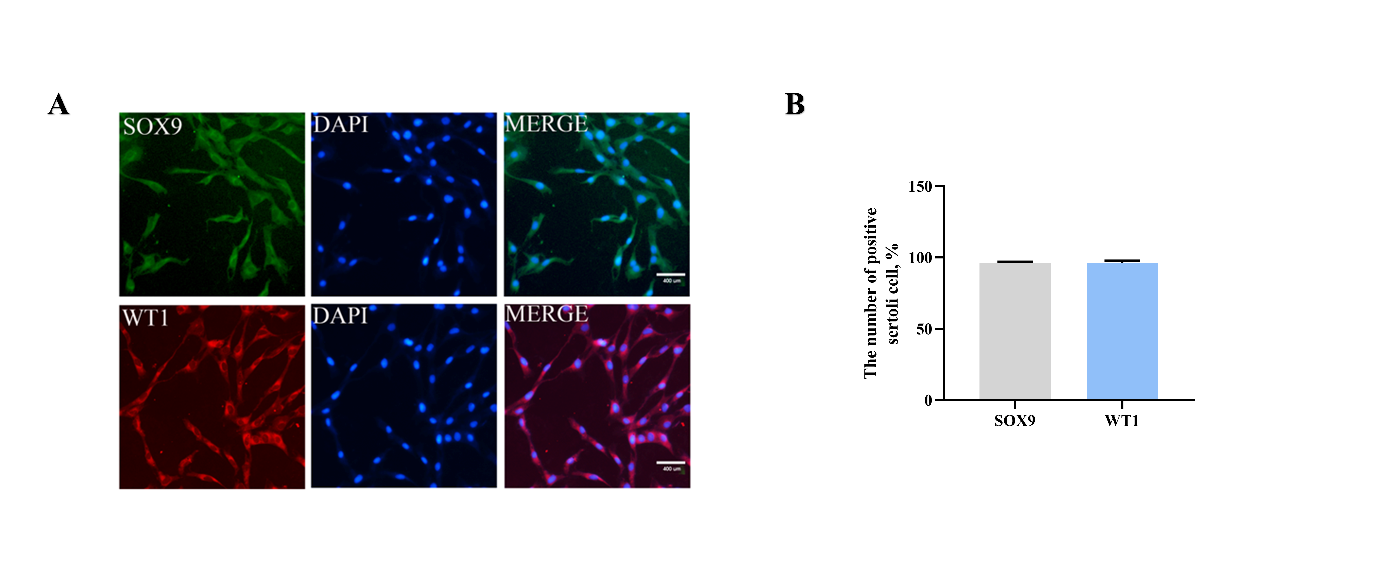
**

**Fig. S1** Isolation and characterization of primary SCs in goats. **A** Identification of isolated primary SCs by IF staining. Bar = 400 µm. **B** Histogram shows the number of positive cells after staining


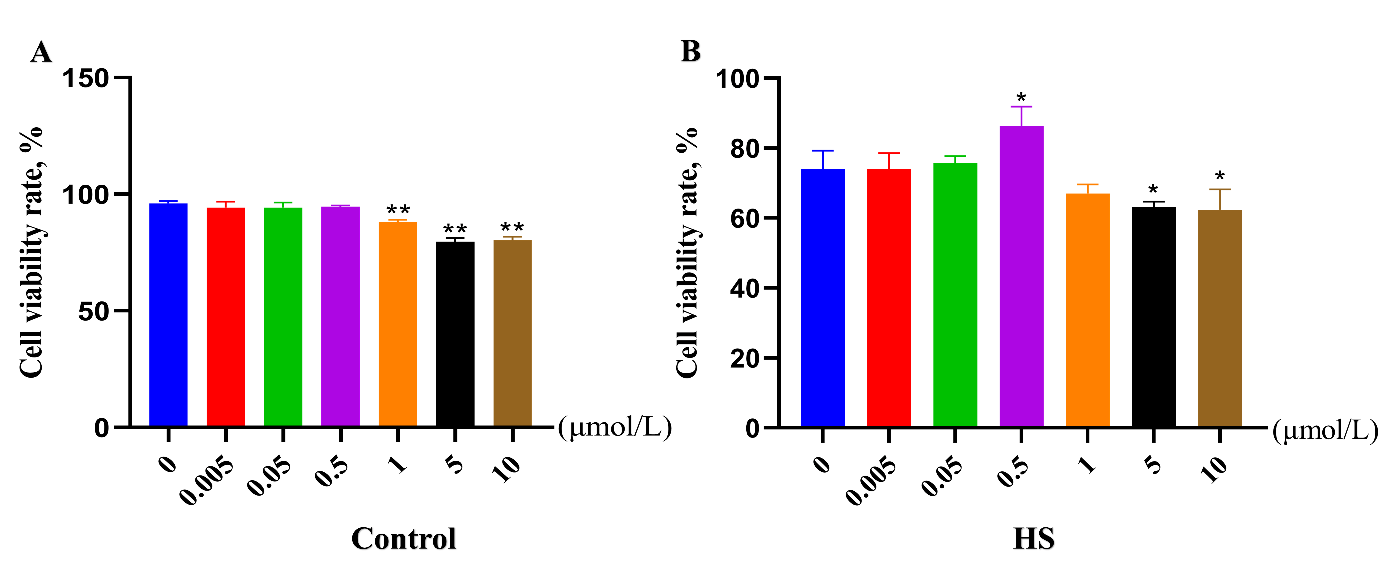


**Fig. S2** Effect of different concentrations of melatonin treated for 24 h on SCs viability. **A** Normal conditions. **B** HS conditions. **P* < 0.05, ***P* < 0.01vs. Control


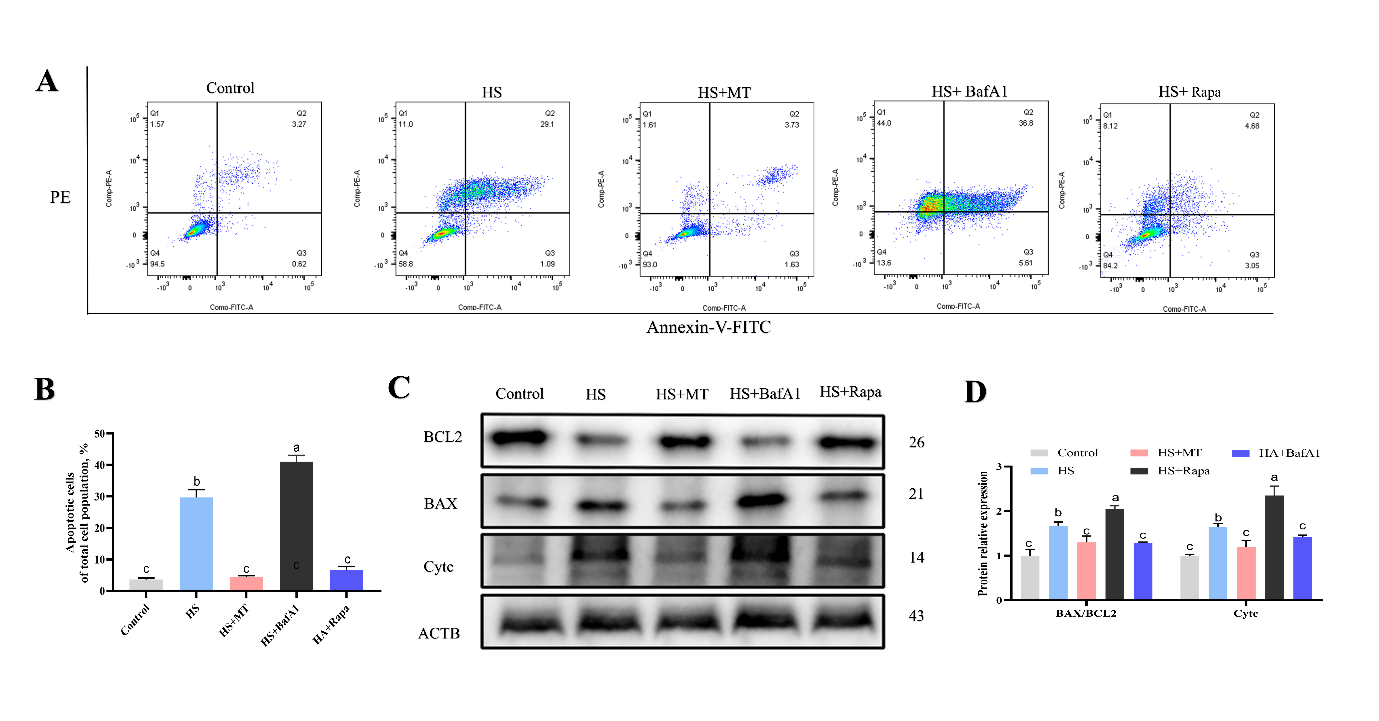


**Fig. S3** The effects of inhibiting or promoting mitophagy on HS-induced apoptosis in SCs. **A** Analysis of SCs apoptosis using flow cytometry. **B** Quantification of flow cytometry data. **C** Expression of mitochondria-dependent apoptotic proteins. **D** Quantification of mitochondria-dependent apoptotic proteins. Note: different letters indicate significant difference
